# FACS-Based Assessment of Human Hematopoietic Stem and Progenitor Cells

**DOI:** 10.3390/ijms26178381

**Published:** 2025-08-28

**Authors:** Tessa Schmachtel, Halvard Bonig, Michael A. Rieger

**Affiliations:** 1 Department of Medicine, Hematology/Oncology, Goethe University Hospital, 60590 Frankfurt, Germany; schmachtel@med.uni-frankfurt.de; 2 Institute for Transfusion Medicine and Immunohematology, Goethe University Frankfurt, 60528 Frankfurt am Main, Germany; h.boenig@blutspende.de; 3 Frankfurt Cancer Institute, 60596 Frankfurt, Germany; 4 German Cancer Consortium, German Cancer Research Center, 69120 Heidelberg, Germany; 5 Excellence Cluster Cardio-Pulmonary Institute, 60590 Frankfurt, Germany

**Keywords:** HSPCs, flow cytometry, cell sorting, single cell

## Abstract

The existing heterogeneity of the human hematopoietic stem cell (HSC) compartment imposes significant challenges in understanding their physiology and molecular constitution. The hematopoietic system is hierarchically organized, with HSCs at the apex, responsible for maintaining homeostasis by ensuring a life-long supply of blood cells. HSCs are highly potent but rare, making their pure isolation challenging. To address this, flow-cytometry-based methods are commonly used to isolate HSCs, bridging the gap between surface marker expression and understanding their functional and molecular properties. However, detailed methodology papers providing practical guidance for the prospective isolation of distinct human hematopoietic stem and progenitor cell (HSPC) populations are rare, hindering reproducible applications across different research groups. Here, we present a comprehensive protocol for isolating multipotent long-term repopulating HSCs (LT-HSCs) and define multipotent progenitor populations (MPPs) from human mobilized peripheral blood (mPB) after leukapheresis using fluorescence-activated cell sorting (FACS). By highlighting the workflow, outlining critical considerations and emphasizing recent advancements in the field, we provide an extensive overview of FACS-based human HSC isolation. This facilitates the enrichment of these rare cells for downstream analysis and enables researchers to improve our understanding of the heterogeneity within the HSC compartment.

## 1. Introduction

The human hematopoietic system is a highly regenerative organ that gives rise to approximately one trillion cells every day. Blood cells are instrumental for oxygen supply, wound healing and immune responses. Tissue surveillance, repair and resilience are dependent on blood cells. To accomplish these life-critical functions, hematopoiesis must be highly dynamic. The life-long blood formation is maintained by hematopoietic stem cells (HSCs) residing at the apex of the hematopoietic differentiation hierarchy [1,2]. Despite an enormous cellular output, the number of HSCs remains low, ranging from 50,000 to 200,000 HSCs during steady-state hematopoiesis [3]. Two key features of HSCs are their self-renewal and multilineage reconstitution potential. Through a stepwise differentiation process, HSCs give rise to various multipotent progenitor (MPP) populations, which successively lose their multilineage potential and become restricted to a distinct lineage while maintaining their own stem cell pool [4,5,6,7]. Conceptually, HSCs can be defined as multipotent long-term repopulating hematopoietic stem cells (LT-HSCs) based on their ability to be serially transplantable into myeloablated recipients and their multilineage and long-term reconstitution potential [8,9,10,11]. These unique characteristics highlight LT-HSCs for therapeutic applications in regenerative medicine and cell and gene therapy. In 1957, E.D. Thomas and his team achieved the first successful hematopoietic stem cell transplantation (HSCT). They treated a leukemia patient with high doses of radiation, followed by the intravenous infusion of bone marrow cells from his identical twin. [12]. Over the following decades, major improvements have been made, and HSCTs are nowadays a cornerstone in the treatment of hematologic malignancies and diseases [13,14,15].

The prospective separation of defined HSPC populations based on phenotypic cell surface markers, known as clusters of differentiation (CD), marked a significant advancement in the field [16]. In 1988, Weismann and colleagues successfully isolated murine HSCs using fluorescence-activated cell sorting (FACS) based on surface markers [4]. This method has since dramatically improved the identification and isolation of murine HSCs through refined cell surface characterization and allows us to link molecular and functional properties of single cells [17,18].

In contrast to the murine hematopoietic system, the prospective isolation of *bona fide* human HSCs requires further refinement. Human HSPCs, including the true HSCs, express the surface marker CD34, and about 0.2–3% of the nucleated cells in the BM are CD34-positive [19,20,21]. Importantly, the vast majority of CD34+ cells are not stem cells with life-long reconstitution ability but have undergone lineage-restricting initial differentiation [16,22]. To further enrich HSCs, additional surface markers must be considered. Over the past decades, numerous publications have contributed to enhancing the purity of the HSC population. For instance, colony-forming assays performed with CD34+CD38- cells demonstrated a homogeneous formation of blast colonies while lacking differentiation marker expression, thereby enriching stem cell potential [23]. The identification of CD90 (Thy1) further improved the isolation of HSCs from the CD34+CD38- compartment [24]. Additional studies concluded that long-term reconstituting cells could be defined as lin-CD34+CD38-CD45RA-CD90+ and distinguished HSCs from multipotent progenitors [17,25,26,27,28]. Even though murine markers can often not be directly transferred to human population, the interaction of murine HSCs with the BM niche through integrin expression gave valuable insights into the key features maintaining quiescence in murine HSCs [29]. Hypothesizing that integrin expression would also mark human HSCs, Dick and colleagues examined the expression of integrins between the CD90+ and the CD90- compartment. CD49f+ expressing CD34+CD38-lin- cells showed a sevenfold increased engraftment in transplanted NSG mice [30]. Nowadays, a combination of these surface markers should be used to prospectively isolate LT-HSCs (defined as lin-CD34+CD38-CD45RA-CD90+CD49f+) using fluorescence-conjugated monoclonal antibodies in FACS [31,32,33].

Flow cytometry is a high-throughput quantitative technology for detecting cell phenotypes. It is based on measuring cell characteristics via light scattering and fluorescence emission. The fluorescence signal occurs from an excitation source (e.g., laser or spectral light) that hits the passing fluid stream directing the cells or moving particle that should be analyzed [34]. Most applications analyze cells stained with fluorophore-labeled antibodies against surface proteins. Coming from a first-generation flow cytometer detecting only cell size (Coulter counter) nearly seventy years ago, we are now able to operate up to 60 detection channels simultaneously using spectral flow cytometry [35,36,37,38,39,40]. Therefore, it becomes increasingly critical to set the right parameters, accurately compensate for the overlapping emission spectra of different fluorochromes within one staining mixture and carefully analyze the acquired data to ensure the optimal design and execution of a FACS experiment.

## 2. Experimental Design

**Figure 1:**
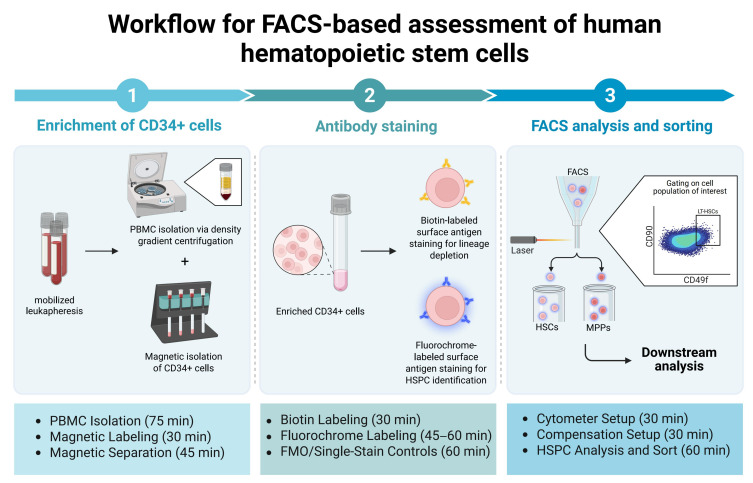
Schematic overview of the experimental workflow used in this protocol. Mononuclear cells were isolated from mobilized leukapheresis products via density gradient centrifugation. CD34^+^ cells were enriched using magnetic-activated cell sorting (MACS), followed by fluorescence-activated cell sorting (FACS). Cells were stained with biotin-labeled antibodies for lineage depletion and fluorochrome-conjugated antibodies for hierarchical gating. Distinct marker combinations were used to isolate hematopoietic stem cells (HSCs) and multipotent cells (MPPs) from hematopoietic stem and progenitor cells (HSPCs) for downstream analysis. This figure was created in BioRender. Rieger, A. (2025). https://BioRender.com/67c7l8l (accessed on 24 August 2025).

This protocol describes the prospective purification of human HSPC subpopulations including the HSC-enriched fraction. Our method utilizes mobilized CD34+ cells from leukapheresis products (mob LPs) of donors treated with granulocyte colony-stimulating factor (G-CSF). Nowadays, these samples represent the main source for allogeneic transplantation due to easier sample collection and an enriched number of CD34+ HSPCs compared to BM aspirations. Additionally, HSCTs performed with mobilized peripheral blood (mPB) samples show identical overall survival rates in the clinics but decreased relapse incidents compared to BM transplantations [41,42,43]. Here, we provide a detailed workflow, including the steps for isolating nucleated cells from fresh and frozen mob LPs, CD34+ cell purification via magnetic cell separation (MACS^®^) and antibody staining for prospective FACS isolation of human HSCs, which can be further processed for subsequent analyses (Figure 1).

## 3. Materials and Equipment

The products shown below are available from various manufacturers at comparable prices. The listed catalog numbers and manufacturers are only a recommendation based on personal experience (Table 1).

Caution:

- Only use polypropylene (PP) tubes for the whole procedure (including FACS tubes). HSCs tend to stick to polystyrene (PS).
- Some staining protocols require commercially purchased staining buffers. These alternatives can be used instead of Horizon™ Brilliant Stain Buffer.
- Alternative products can be purchased from different providers at similar prices. The listed catalog numbers and providers are only a recommendation based on personal experience.
- MACS washing buffer: Dilute MACS^®^ BSA Stock Solution at a ratio of 1:20 with autoMACS^®^ Rinsing Solution; a temperature of 2–8 °C should be maintained.

## 4. Protocol

### 4.1. Enrichment of CD34+ Cells from Leukapheresis Products After Mobilization

This protocol is based on using the FACSAria™ III Cell Sorter (BD) and may differ for other flow cytometers. The leukapheresis samples (mob LPs), each with a volume of approximately 1 mL, are provided as pseudonymized residual material in EDTA tubes. Before enrichment of CD34+ cells, peripheral blood mononuclear cells (PBMCs) are isolated from these samples using density gradient centrifugation.

#### 4.1.1. Isolation of PBMCs from Fresh Leukapheresis

1. Dilute the leukapheresis products with PBS (1×) at ratios of 1:1 or 1:2.
2. Aliquot 3 mL of Pancoll into a 15 mL falcon tube and bring to room temperature (RT).
3. Top 3 mL aliquoted Pancoll with cell suspension.
4. Centrifuge at 400× *g* for 30 min at RT without break.
5. Isolate PMBCs from the interphase between the plasma and Pancoll layer. Transfer the interphase into a fresh 15 mL falcon tube.
6. Wash isolated PMBCs 2 times with 5–7 mL PBS (1×) for 10 min at 200× *g*, 20 °C.
7. Resuspend washed mononuclear cells in 5 mL MACS^®^ washing buffer. Take 10 µL of the cell suspension to determine cell number. Centrifuge PMBCs again for 7 min at 300× *g*, 4 °C.
8. Resuspend cells in 300 µL MACS^®^ washing buffer for up to 10^8^ cells (if this cell number is exceeded, increase the reagent volume for magnetic cell labeling).

Caution:

- During density gradient centrifugation, it is crucial to avoid mixing the layers of the leukapheresis product and Pancoll solution. This careful separation allows the effective isolation of lymphocytes and monocytes from other types of blood cells. During centrifugation, erythrocytes are aggregated by polysucrose and rapidly sediment, whereas mononuclear cells remain at the plasma interphase. Erythrocytes pellet to the bottom of the centrifuge tube. Due to their higher density, granulocytes will not remain in the interphase and will move toward the bottom of the centrifuge tube.
- Centrifugation below RT can lead to impaired separation of macromolecules in the leukapheresis sample.
- After density gradient separation, the isolated PBMC solution will contain platelets. Washing at low speed, 20 °C for 10 min, helps remove platelets from cell suspension and increase purity.
- Cell number should be obtained manually via microscopy. We recommend counting cells in a Neubauer chamber. For this protocol, 10 µL of cell suspension is mixed with 10 µL of trypan blue (diluted at 1:1 with 1× PBS). C-Chips are loaded with 10 µL of pre-mixed cell/trypan blue solution.

#### 4.1.2. Processing of Frozen PBMCs

1. Thaw the previously isolated and frozen PBMCs from leukapheresis (mob LPs) by immersing the vials in a 37 °C water bath.
2. Transfer completely thawed cells into a 15 mL falcon tube filled with 10 mL of thawing media at RT.
3. Centrifuge cells at 300× *g* for 7 min at RT.
4. Resuspend cells in 10 mL StemStan™ SFEM II media and wash again at 300× *g* for 7 min at RT.
5. Determine cell number and viability and resuspend in appropriate volume of ml MACS^®^ washing buffer.

#### 4.1.3. Magnetic Labeling and Separation of Isolated PBMCs

Magnetic Labeling

1. To obtain pre-separation single-cell solution, pass cells through 30 µm nylon mesh.
2. Add 100 µL of FcR Blocking Reagent.
3. Add 100 µL of CD34 MicroBeads UltraPure.
4. Mix the solution and incubate for 30 min at 4 °C.
5. Wash cells with 5–10 mL buffer, centrifuge for 10 min at 300× *g*, 4 °C.
6. Resuspend cells in 500 µL buffer for up to 10^8^ cells.

Caution:

- Avoid air bubbles, since they can block the column.
- Pre-separation filtering removes cell clumps and debris. The single-cell solution increases labeling performance and prevents clogging of columns during magnetic separation.
- FcR blocking reagent is added to avoid unwanted binding of antibodies to human Fc receptors, which would lead to unspecific signals in flow cytometry.
- If cell number exceeds 10^8^ cells, increase the reagent volume for magnetic cell labeling according to the manufacturer’s protocol.

Magnetic Separation

1. LS columns are placed in MACS^®^ Quadro Separator.
2. Prepare the column by rinsing it with 3 mL MACS^®^ washing buffer.
3. Filter the samples again through 30 µm pre-separator filter to remove aggregated cells and prevent clogging of columns.
4. Apply 5 mL cell suspension onto the column. Collect the flow-through containing unlabeled cells.
5. Wash magnetically bound cells in the column by applying 3 × 3 mL of MACS^®^ washing buffer.
6. Remove the column from the separator and place it into 5 mL PP tube.
7. Add 3–5 mL of MACS^®^ washing buffer. Immediately flush out the magnetically labeled cells by firmly pushing the plunger into the column.
8. Take 100 µL aliquot as unstained control for cytometer and FACS analysis.
9. Determine cell number and centrifuge cells at 300× *g* for 7 min.

Caution:

- The required rinsing volume varies according to the specific column and MACS Separator employed. Use an appropriate amount and adapt volumes and consumables according to your experimental layout.
- Purity can be increased by repeating the separation procedure with eluted fraction.
- Unstained cells function as setup control for adjusted photomultiplier tube (PMT) voltages.

### 4.2. Antibody Staining

#### 4.2.1. Biotin-Labeled Antibody Staining of CD34+ HSPCs

1. Resuspend cells in 50 µL MACS^®^ buffer. Purified CD34+ cells are stained with biotin-labeled antibodies against lineage markers CD2, CD3, CD14, CD16, CD19, CD56 and CD235a to exclude lineage-positive cells (Table 2).
2. Incubate cells for 15 min in a refrigerator.
3. Wash cells with 1× PBS via centrifugation at 300× *g* for 7 min at 4 °C to remove unbound antibody.
4. Discard the supernatant and resuspend cells in 50 µL of Horizon™ Brilliant Stain Buffer for fluorochrome-labeled antibody staining.

#### 4.2.2. Fluorochrome-Labeled Antibody Staining of CD34+ HSPCs

1. Stain 50 µL of purified CD34+ cells with fluorochrome-labeled antibody mixture (Table 3).
2. Mix cells by gently pipetting the solution up and down at least 5 times.
3. Incubate the cell solution for 30 min on ice without light exposure.
4. Wash cells with 3–5 mL of 1× PBS via centrifugation at 300× *g* for 7 min at 4 °C to remove unbound antibodies.
5. Remove the supernatant and resuspend cells in 500 µL of MACS^®^ buffer.
6. The samples are ready for analysis in flow cytometry.

Caution:

- The exposure of light leads to degradation of the fluorochromes and decreased signals for the following experimental analysis. Incubating cells in the dark is therefore recommended.
- Antibody volumes are calculated for 5 × 10^6^ cells. For increased cell numbers, increase the indicated volumes accordingly.
- It is advisable to use fluorochromes with strong fluorescence intensities for detecting antigens with low expression levels while reserving fluorochromes with lower fluorescence intensities for markers with high expression levels.
- Titration of the new antibodies is needed prior to the experiment. This helps optimize the concentrations and combinations of conjugated antibodies.
- Some staining protocols require commercially purchased staining buffers. These alternatives can be used instead of the Horizon™ Brilliant Stain Buffer.

#### 4.2.3. Fluorescence-Minus-One (FMO) Controls for Gate Setting

1. Label the FACS tubes for each FMO control, as illustrated in Table 4.
2. Aliquot 1 × 10^5^ CD34+ cells into each labeled FACS tube.
3. Add 1 μL of each antibody conjugate to the associated labeled FACS tube.
4. Mix the FMO controls by vortexing and incubate tubes for 30 min on ice without light exposure.
5. Add 3–5 mL of PBS (1×) and centrifuge tubes at 300× *g* for 7 min.
6. Discard the supernatant and resuspend FMO controls in 200 µL MACS^®^ buffer.
7. The FMO controls are ready for analysis in the cytometer.

Caution:

- This step may not be required once the panel has been established and validated.
- FMO controls can assist in setting gates, but they should not be used exclusively for defining gates in FACS experiments. Relying strictly on FMO controls for gating can result in the exclusion of antigens that are expressed at low levels.

#### 4.2.4. Single Antibody Stain Control for Compensation

1. Experiments involving multiple parameters can lead to spectral spillovers of a fluorochrome into another detector. To prevent false positive signals caused by spectral spillover, single-antibody-stained controls should be used for compensation setup.
2. Label the FACS tubes for each antibody conjugate that will be used in the experiment, as illustrated in Table 5.
3. Mix the compensation beads by vigorously inverting at least 10 times or vortexing.
4. Aliquot one drop of compensation beads into each labeled FACS tube.
5. Add 1 μL of each antibody conjugate to the respective FACS tube.
6. Mix the single staining solution by vortexing and incubate tubes for 20 min on ice without light exposure.
7. Add 3–5 mL of PBS (1×) and centrifuge tubes at 300× *g* for 7 min.
8. Discard the supernatant and resuspend single staining solutions in 200 µL MACS^®^ buffer.
9. The compensations are ready for analysis in the cytometer.

Caution:

- Single-cell staining controls for compensation can also be performed using cells that positively express the relevant antigens to determine spectral overlap. If the cells of interest do not express the antigen, compensation beads are a suitable alternative.
- Single antibody staining for viability dye compensation should be performed using cells. A positive population of dead cells can be generated by inducing cell death through heat shock.

### 4.3. FACS Analysis and Sorting

#### 4.3.1. Setting up FACS Machine

1. The workflow outlined in this protocol details the process of sorting LT-HSCs and MPPs from human mob LPs using the FACSAria™ III Cell Sorter (BD) and is based on the manufacturer’s manual. Other FACS instruments with similar configurations may also be appropriate.
2. Fluidics startup is performed according to cytometer instructions.
3. Sorting human HSPCs can be performed using a 70 µm nozzle.
4. Turn on the stream and optimize the breakoff by adjusting the amplitude. The gap size adjusts automatically according to your set amplitude. For performing sorts using the 70 µm nozzle, the gap size should be valued around 6–7.
5. Run a CST performance check before every sort by using BD^®^CS&T Beads (BD). Laser delay depends on the daily performance of the lasers and can vary from day to day.
6. Turn on the sweat spot to enable stable stream.
7. Set the drop delay manually or automatically using BD^®^ FACS Accudrop technology. The drop delay is the time interval required for the drop drive energy to be applied to the stream, breaking it into highly uniform droplets containing the particles of interest, and it is crucial for efficient sorting.
8. Adjust photomultiplier tubes (PMTs) to an autofluorescent area by using the unstained control. The FACSAria™ III (BD) uses PMTs to convert scattered light and all fluorescence channels into an electrical signal called pulse. To optimize the PMT settings, unstained cells can be used to detect the levels of autofluorescence.
9. The fluorescent signals are measured on a five-decade log scale; therefore, stained cells can be up to five decades brighter than unstained cells. Single-antibody-stained controls indicate the detection range of the fluorescent signal and are used to adjust PMTs.
10. Adjust the side streams, so that cells gently slide into the sorting buffer. Directly hitting the liquid surface increases the risk of cell death.

Caution:

- The 70 µm nozzle is appropriate for cells < 12 µm in size. If working with larger or fragile cells, a larger nozzle size and lower pressure are recommended to exclude the risk of exposing cells to excessive shear stress.
- Check the deflection plates before turning on the stream; residual salts can affect the sorting performance.
- Modern cell sorters can measure signals across up to a seven-decade log scale. Adjust the fluorescent signals according to your specific cytometry setup.

#### 4.3.2. Gating Strategy for Discriminating Human MPPs and HSCs

**Figure 2:**
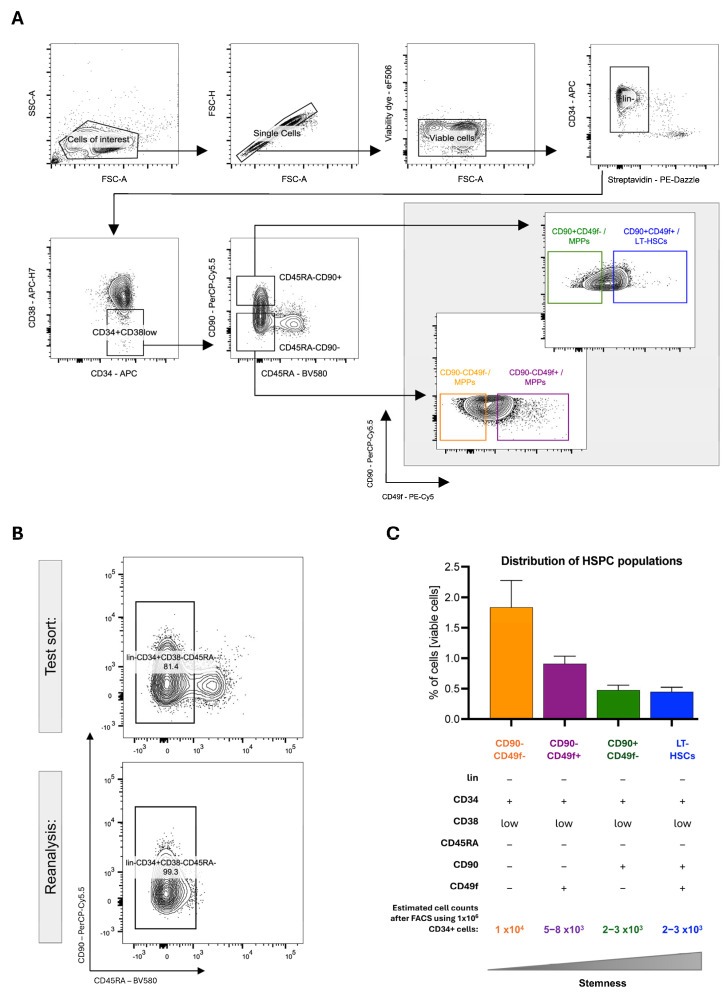
**Sorting strategy for isolation of human HSPCs**: (**A**) Hierarchical gating of human HSPCs. (**B**) Reanalysis of sorted cells to detect sorting efficiency. (**C**) Quantitative distribution of HSPC populations calculated from viable cells (*n* = 15).

1. After setting up the cytometer, a gating strategy must be established. This involves using single antibody controls to compensate for spectral spillover and FMO controls to set gate boundaries. The hierarchical gating strategy depends on the experimental layout. Figure 2A shows the hierarchical gating strategy of the analysis template displaying consecutive 2D FACS contour-dot plots. The individual FACS plots of the template are shortly explained here.
2. **Cells of interest**: Forward scatter (FSC-A) and sideward scatter (SSC-A) are used to exclude debris on a linear scale. The gate can be established by measuring the unstained control.
3. **Singlets:** Doublets causing unspecific signals can be excluded by analyzing the samples in FSC-Area (FSC-A) scaling versus FSC-Height (FSC-H) scaling.
4. **Viable cells**: Dead cells are excluded by a viability gate.
5. **Lin-** (CD2-, CD3-, CD14-, CD16-, CD19-, CD56- and CD235a-): Exclusion of the remaining lineage-positive progenitors is achieved via streptavidin–secondary antibody recognition of biotinylated antibodies.
  - **Lin-CD34+CD38low**
  - **Lin-CD34+CD38lowCD45RA-CD90-**
  - **Lin-CD34+CD38lowCD45RA-CD90+**
6. HSPC populations including LT-HSCs can be identified using specific marker combinations adapted from the publication of Kaufmann et al. (Figure 2A) [44]:
  - **CD90-CD49f-/MPPs** (Lin- CD34+CD38-CD45RA-CD90-CD49f-),
  - **CD90-CD49f+/MPPs** (Lin- CD34+CD38-CD45RA-CD90-CD49f+),
  - **CD90+CD49f-/MPPs** (Lin- CD34+CD38-CD45RA-CD90+CD49f-),
  - **CD90+CD49f+/LT-HSCs** (Lin- CD34+CD38-CD45RA-CD90+CD49f+).

#### 4.3.3. FACS Sorting of Human MPPs and LT-HSCs

1. Once the hierarchical gating is established, cells can be sorted.
2. For sorting two or more populations, the sort precision mode should be set to “Purity” or “4-Way Purity”.
3. Reanalysis of the sorted cell populations is essential to ensure reliable cell separation. Sufficient cell numbers for reanalysis can be obtained by using a more abundant cell population higher up in the hierarchy; therefore, Lin-CD34+CD38low/-CD45RA- are used to validate the sort efficiency and cytometer settings. The sorting purity should be >95% (Figure 2B).
4. After sorting MPPs and LT-HSCs, viable cells should be counted microscopically and can be processed for downstream analysis.
5. Data analysis can be performed using Diva 8.1 or FlowJo software (BD).

Caution:

- The yield mask, purity mask and phase mask settings address different types of conflicts during sorting. These settings work together to define the sort precision modes, which are detailed in the manufacturer’s manual.
- The recommended flow rate should not exceed 8000–10,000 events per second, as higher flow rates can increase the risk of nozzle O-ring occlusion and decrease sorting efficiency.
- During reanalysis, the sorted cells may have lost signal intensities and are localized near the boundaries of the set gates due to fluorochrome bleaching. Therefore, correctly sorted cells can fall outside the initial gates, necessitating slight gate adjustments to account for bleaching effects.

## 5. Expected Results and Limitations

Isolating LT-HSCs and MPPs from mobilized leukapheresis products typically results in a post-sort viability of approximately 70–80%, as indicated by FACSAria™ III Cell Sorter (BD) reports. The relative distribution of different HSPC populations can vary considerably between samples, and the overall frequency of HSPCs in CD34-enriched fractions generally remains below 2%. Consequently, the absolute numbers of LT-HSCs and MPPs obtained after FACS isolation are often limiting for downstream analyses. For example, processing 1 × 10^6^ CD34^+^ HSPCs by FACS typically yields around 2000–3000 LT-HSCs and approximately 10,000 MPPs (Figure 2C). Therefore, depending on the requirements of downstream applications, multiple samples may need to be processed to obtain sufficient cell numbers. It should be mentioned that while we provide a detailed established protocol here, the manuscript format does not entail the results of validation experiments.

It is important to note that the sorting procedure imposes significant mechanical and oxidative stress on the cells, which can induce their activation and affect cell viability. As a result, isolated LT-HSCs and MPPs rapidly lose their quiescent state and begin to differentiate into more restricted progenitors within 24 h of in vitro culture [45]. To preserve their functional properties, assays such as colony-forming unit (CFU) assays or single-cell tracking should be initiated immediately after isolation. Moreover, the protocol steps should be adapted according to the intended downstream application. For example, if the sorted cells are to be used for in vivo transplantations in immunocompromised NSG mice, they should be collected directly into an appropriate volume of PBS for subsequent procedures. In contrast, when preparing FACS-sorted cells for single-cell sequencing experiments, the cells are collected in BD Pharmingen™ stain buffer. In our laboratory, we employ the BD Rhapsody system, where cells are first subjected to sample tag and fluorescence-labeled antibody staining and then sorted and pooled. Afterward, the sorted cell pool is stained with oligonucleotide-labeled antibodies (AbSeq) for single-cell capture and subsequent library preparation. For bulk molecular analyses of the sorted cells (transcriptomic, epigenetic or proteomic analyses), cells are directly sorted into small volumes of appropriate lysis buffer (10–50 µL) in Eppendorf or PCR tubes.

The instrument setup and technical expertise are also critical for successful cell isolation via FACS, particularly when targeting complex subpopulations. Designing effective multicolor panels requires meticulous planning and extensive testing, including careful optimization of marker selection, antigen expression combinations, antibody clones and fluorochrome choices, all tailored to the specific experimental setup and cytometer availability. Spectral overlap in multicolor antibody panels demands precise compensation to accurately distinguish different cell populations. Sorting speed must be carefully balanced: while high-speed sorting can compromise cell viability and purity, slower sorting increases the risk of contamination, especially if the cytometer is not operated in a sterile environment. Additionally, laser exposure during sorting can cause fluorochrome bleaching, reducing the signal intensity of reporters and potentially leading to misidentification of target cells. Furthermore, the quality of the sample significantly affects cell viability and subsequent analysis. To minimize stress on the cells, fresh samples should be processed immediately. Freezing and thawing can greatly diminish cell survival rates, and outcomes are highly dependent on the techniques used for cryopreservation.

By taking these factors into consideration, researchers can overcome existing challenges in the field of human HSC isolation and leverage the technical advancements available today to enhance our understanding and capabilities in this area and successfully obtain high-quality, functionally relevant stem cell populations for downstream applications.

## 6. Discussion

The purification of a homogenous HSC population remains critical for accelerating our understanding of stem cell physiology. Traditionally, HSC biology has been studied by focusing on prospectively isolating cells with higher stem cell potential and investigating their molecular and functional characteristics using single-cell approaches. Therefore, researchers use defined cell populations, which can be consistently isolated across different laboratories. Here, we describe a highly reproducible method for isolating human LT-HSCs and MPP populations from mob LPs using key surface markers (CD34, CD38, CD45RA, CD90, CD49f) and a hierarchical gating strategy. This sorting strategy has been successively refined by numerous groups [30,46,47,48].

Despite the substantial progress researchers have achieved in understanding human HSC physiology and biology at the functional and molecular level, critical challenges remain to be solved. First, nowadays, sorting protocols, including the one shown here, can only enrich for human HSCs, but they still fail to absolutely purify them due to remaining heterogeneity. Second, the common procedures used to identify and sort for defined HSPC populations from different sources vary between laboratories and hinder direct comparisons of the results. Third, the read-out of functional properties, including the true stemness of sorted cells, depends on the chosen xenograft model and procedure of transplantation. Fourth, robust culture protocols for maintaining and expanding human HSCs are still under development.

Depending on the utilized source material, isolated HSCs can highly differ in their stem cell composition and engraftment capabilities. Therefore, the pursuit of a purer HSC population across different adult hematopoietic tissues has progressed slowly. The difficulty in obtaining primary human samples and maintaining robust scientific platforms has further compounded these challenges. Recent studies mainly implement CB samples instead of adult tissues. Therefore, one should emphasize the need to determine whether current surface antigens can delineate similar HSC-enriched populations using mPB, mLP or BM samples [27,49,50].

In addition to these recent improvements in prospective isolation of HSCs, functional read-outs of phenotypically defined populations must be evaluated with caution. A significant challenge in assessing the potency of human HSCs is their functional evaluation in xenograft models. The current gold standard involves the use of non-obese diabetic/severe combined immunodeficient (NOD/SCID) mice with an *IL2*Rγ^null^ mutation, commonly referred to as NSG mice [51,52,53,54]. These xeno-tolerant mouse strains, when transplanted with human HSCs, can simulate the human immune system but still harbor major genetic differences and are deficient in the reconstitution of specific blood cells, such as granulocytes, platelets and erythrocytes [55]. To overcome these obstacles, researchers developed the NSGW41 mouse strain, enabling improved human erythropoiesis and platelet formation, and demonstrated significant advantages in HSC engraftments due to the preferred expansion of *KIT*-sufficient donor cells in the BM without previous irradiation conditioning of the recipients [56,57]. Nevertheless, NSG and NSGW41 models remain limited in low-cell transplantations and do not capture the pathogenesis of graft-versus-host disease (GvHD), a major cause of non-relapse mortality following allogeneic HSCT [58]. While xenograft models offer significant advantages by bridging the gap between preclinical mouse models and clinical trials, they require further refinement to fully elucidate the complexities of HSC biology.

Along with employing suitable in vivo models for testing HSC potency, researchers must understand the core stem cell networks that govern HSC self-renewal, as demonstrated in the groundbreaking work with embryonic and induced pluripotent stem cells [59]. Utilizing mechanisms that maintain HSCs would enable us to expand them in culture while maintaining their ability to reconstitute multiple lineages for advanced cell and gene therapy approaches. Despite the significant advances made in extending the culture period, such as the introduction of UM171 and polyvinyl alcohol (PVA)-based culture conditions, long-term culture remains variable due to cell quality and HSC intrinsic heterogeneity [60,61,62,63].

In recent years, developments in single-cell technologies, e.g., single-cell cellular indexing of transcriptome and epitope (CITE) sequencing, have been able to significantly enhance our understanding of HSC characteristics [64]. The high-resolution analysis on transcriptomic and proteomic level allows us to gain insights into cellular heterogeneity to refine the sorting strategies of marker-defined HSPC populations [65,66,67,68,69]. By using this technology, Komic, et al. generated a comprehensive molecular map of early human hematopoietic differentiation and identified a distinct subpopulation of immature quiescent HSPCs characterized by high expression of CD273/PD-L2, modulating the immune responses of T cells [70]. Another study conducted by Anjos-Afonso et al. was able to approve their newly defined HSC subpopulation expressing the endothelial protein C receptor (EPCR) as homogenous stem cell population via single-cell transcriptomic analysis. In this study, hierarchical gating on lin-CD34+CD38-CD45RA-CD49f+ from CB and BM samples enabled a stem cell frequency of approximately one in three cells [71].

In summary, while HSCs are the best studied somatic stem cells due to the ability to prospectively isolate these cells, considerable heterogeneity remains, and major challenges must be overcome. To fully comprehend the mechanisms maintaining HSCs in an undifferentiated state, researchers must embark on an extensive exploration, investigating numerous intrinsic and extrinsic factors and their impacts on HSC function and behavior. Additionally, factors such as aging, stress, inflammation and other pathophysiological events, while not discussed here, further contribute to HSC heterogeneity. Therefore, ongoing exploration is essential for advancing our understanding of human HSC biology, particularly in the context of clinical cell therapy and future applications in regenerative medicine.

## Figures and Tables

**Table 1:** Reagents, consumables and equipment.

Consumables	Catalog Number	Company
Anti-Human CD14 [61D3]	13-0149-82	Thermo Fisher Scientific, Waltham, MA, USA
Anti-Human CD16 [CB16]	13-0168-82	Thermo Fisher Scientific, Waltham, MA, USA
Anti-Human CD19 [HIB19]	13-0199-82	Thermo Fisher Scientific, Waltham, MA, USA
Anti-Human CD2 [RPA-2.10]	13-0029-82	Thermo Fisher Scientific, Waltham, MA, USA
Anti-Human CD235a [HIR2]	13-9987-82	Thermo Fisher Scientific, Waltham, MA, USA
Anti-Human CD3 [OKT3]	13-0037-82	Thermo Fisher Scientific, Waltham, MA, USA
Anti-Human CD34 [8G12]	345804	BD Bioscience, Frankling Lakes, NJ, USA
Anti-Human CD38 [HB7]	656646	BD Bioscience, Frankling Lakes, NeJ, USA
Anti-Human CD45RA [HI100]	304132	BioLegend, San Diego, CA, USA
Anti-Human CD49f [GoH3]	551129	BD Bioscience, Frankling Lakes, NJ, USA
Anti-Human CD56 [NCAM]	13-0567-82	Thermo Fisher Scientific, Waltham, MA, USA
Anti-Human CD90/Thy1 [5E10]	561557	BD Bioscience, Frankling Lakes, New Jersey, USA
autoMACS^®^ Rinsing Solution	130-091-222	Miltenyi Biotec, Bergisch Gladbach, Germany
BD^®^ Accudrop Beads	345249	BD Bioscience, Frankling Lakes, NJ, USA
BD^®^ CS&T Research Beads	655051	BD Bioscience, Frankling Lakes, NJ, USA
C-Chip, Neubauer Improved Counting chamber	PK36.1	Roth, Karlsruhe, Germany
CD34 MicroBead Kit UltraPure human	130-100-453	Miltenyi Biotec, Bergisch Gladbach, Germany
Cellstar^®^ sterile centrifuge tubes (15 mL)	188271	Greiner Bio-One, Frickenhausen, Germany
Centrifuge Rotana 460R	5650SET1	Hettich, Tuttlingen, Germany
eBioscience™ UltraComp eBeads	01-2222-42	Thermo Fisher Scientific, Waltham, MA, USA
FACSAria™ III Cell Sorter		BD Bioscience, Frankling Lakes, NJ, USA
FACSDiva™ Software (version 8.1)		BD Bioscience, Frankling Lakes, NJ, USA
Fixable Viability dye	65-0866-14	Thermo Fisher Scientific, Waltham, MA, USA
FlowJo™ Software (version 10.10.0)		BD Bioscience, Frankling Lakes, NJ, USA
Gibco™ 1× PBS	633801	Thermo Fisher Scientific, Waltham, MA, USA
Horizon™ Brilliant Stain Buffer	563794	BD Bioscience, Frankling Lakes, NJ, USA
LS Columns	130-042-401	Miltenyi Biotec, Bergisch Gladbach, Germany
LSRFortessa™ Cell Analyzer		BD Bioscience, Frankling Lakes, NJ, USA
MACS^®^BSA Stock Solution	130-091-376	Miltenyi Biotec, Bergisch Gladbach, Germany
Pancoll human, Density: 1.077 g/mL	P04-60500	PAN-Biotech, Aidenbach, Germany
Polypropylene FACS tubes (5 mL)	10171942	Thermo Fisher Scientific, Waltham, MA, USA
Polypropylene round bottom tube (14 mL)	10574991	Thermo Fisher Scientific, Waltham, MA, USA
Polystyrene FACS tubes (5 mL)	551.579	Sarstedt, Nuembrecht, Germany
Polystyrene FACS tubes with cell-strainer cap (5 mL)	10585801	Thermo Fisher Scientific, Waltham, MA, USA
Pre-Separation Filters (70 µm)	130-095-823	Miltenyi Biotec, Bergisch Gladbach, Germany
QuadroMACS™ Separator	130-090-976	Miltenyi Biotec, Bergisch Gladbach, Germany
StemStan™ SFEM II media	9655	STEMCELL Technologies, Vancouver, Canada
Streptavidin	405248	BioLegend, San Diego, CA, USA

**Table 2:** Biotin-labeled antibodies for lineage-marker positive cell depletion.

Antibody [Clone]	Volume for 5 × 10^6^ Cells (Predilution)
CD2 [RPA-2.10]	0.5 µL
CD3 [OKT3]	2.4 µL (1:10)
CD14 [61D3]	0.5 µL
CD16 [CB16]	0.5 µL (1:10)
CD19 [HIB19]	1 µL
CD56 [NCAM]	2.5 µL (1:50)
CD235a [HIR2]	2.5 µL (1:100)

**Table 3:** Fluorochrome-labeled antibody mixture for HSPC classification.

Antibody [Clone]	Fluorochrome	Volume for 5 × 10^6^ Cells
Viability dye	ef506	2 µL
CD45RA [HI100]	BV580	5 µL
CD90/Thy1 [5E10]	PerCP-Cy5.5	5 µL
Streptavidin	PE-Dazzle 594	1.5 µL (of a 1:10 dilution)
CD49f [GoH3]	PE-Cy5	2.5 µL
CD34 [8G12]	APC	2.5 µL
CD38 [HB7]	APC-H7	5 µL

**Table 4:** Staining setup for FMO controls.

Tube Label	Viability Dye eF506	CD45RA BV580	CD90 PerCP-Cy5.5	Strep. PE-Dazzle	CD49f PE-Cy5	CD34 APC	CD38 APC-H7
FMO-eF506	−	+	+	+	+	+	+
FMO-BV580	+	−	+	+	+	+	+
FMO-PerCP-Cy5.5	+	+	−	+	+	+	+
FMO-PE-Dazzle 594	+	+	+	−	+	+	+
FMO-PE-Cy5	+	+	+	+	−	+	+
FMO-APC	+	+	+	+	+	−	+
FMO-APC-H7	+	+	+	+	+	+	−

**Table 5:** Staining setup for compensation controls.

Tube Label	Viability Dye eF506	CD45RA BV580	CD90 PerCP-Cy5.5	Strep. PE-Dazzle	CD49f PE-Cy5	CD34 APC	CD38 APC-H7
eF506	+	−	−	−	−	−	−
BV580	−	+	−	−	−	−	−
PerCP-Cy5.5	−	−	+	−	−	−	−
PE-Dazzle 594	−	−	−	+	−	−	−
PE-Cy5	−	−	−	−	+	−	−
APC	−	−	−	−	−	+	−
APC-H7	−	−	−	−	−	−	+

## Data Availability

The data supporting the findings of this study are available from the corresponding author, MR, upon request.

## Abbreviations

The following abbreviations are used in this manuscript:

CB	Cord blood
CD	Cluster of differentiation
CITE	Cellular indexing of transcriptome and epitope
EPCR	Endothelial protein C receptor
FACS	Fluorescence-activated cell sorting
FMO	Fluorescence-minus-one
G-CSF	Granulocyte colony-stimulating factor
GvHD	Graft-versus-host disease
HSC	Hematopoietic stem cell
HSCT	Hematopoietic stem cell transplantation
HSPC	Hematopoietic stem and progenitor cell
LT-HSC	Long-term repopulating hematopoietic stem cell
mob LPs	Mobilized CD34+ cells from leukapheresis products
mPB	Mobilized peripheral blood
MPP	Multipotent progenitor
NOD/SCID	Non-obese diabetic/severe combined immunodeficient mice
PBMCs	Peripheral blood mononuclear cells
PMT	Photomultiplier tube
PP	Polypropylene
PS	Polystyrene
